# Satisfaction among non-conveyed patients and significant others when discharged at the scene by the ambulance service: an exploratory cross-sectional survey

**DOI:** 10.1186/s12873-022-00659-9

**Published:** 2022-06-07

**Authors:** Glenn Larsson, Alma Dagerhem, Jonas Wihlborg, Andreas Rantala

**Affiliations:** 1 PreHospen-Centre for Prehospital Research, Faculty of Caring Science, Work Life and Social Welfare, University of Borås, Borås, Sweden; 2 Department of Prehospital Emergency Care, Sahlgrenska University Hospital, Gothenburg, Sweden; 3 Emergency Department, Halland Hospital, Halmstad, Sweden; 4 School of Health and Welfare, Dalarna University, Falun, Sweden; 5 Emergency Department, Helsingborg General Hospital, Helsingborg, Sweden; 6 Department of Health Sciences, Lund University, P.O. Box 157, SE-221 00 Lund, Sweden; 7 Centre of Interprofessional Cooperation within Emergency Care (CICE), Linnaeus University, Växjö, Sweden

**Keywords:** Pre-hospital emergency care, Ambulance care, Ambulance services, Nursing, Patient, Significant others, Non-urgent, Non-conveyance, Patient satisfaction

## Abstract

**Background:**

The ambulance service is facing an increased number of calls and ambulance assignments. Between 12 and 42% of all assignments result in non-conveyance to the Accident and Emergency Department. However, there is limited knowledge regarding satisfaction among patients and significant others when patients are assessed as non-urgent and discharged at the scene. Therefore, the aim of the study was to explore and compare satisfaction with the ambulance service among patients and significant others when the patient was discharged at the scene.

**Methods:**

The present study was designed as a cross-sectional exploratory survey with a consecutive sample employing the Consumer Emergency Care Satisfaction Scale questionnaire on patients and significant others.

**Results:**

A total of 162 questionnaires were analysed, 87 patients and 75 significant others. Overall, satisfaction was high with no significant difference between patients and significant others, although 17-19% were dissatisfied with the discharge information.

**Conclusions:**

Generally, patients and significant others are satisfied with the care provided by the Ambulance Service when discharged at the scene and thus not conveyed the Accident and Emergency Department. The participants were especially satisfied with Specialist Ambulance Nurses’ interpersonal skills, e.g., making time and providing thorough information. Guidelines for assignments involving non-conveyance, as well as information, instructions and what to expect when discharged at the scene can be improved.

## Introduction

Mainly due to difficulties with the accessibility of primary care and an aging population, the Ambulance Service (AS) as well as Accident and Emergency departments (A&E) are reporting more presenting patients [1, 2]. Thus, the Ambulance Service (AS) is increasingly employing non-conveyance standards for patients with conditions assessed as non-urgent, thereby attempt to reduce unnecessary visits to the A&E [3]. The phenomenon of non-conveyance has been explored through epidemiological measures [4–8], indicating that between 12 and 42% of all ambulance assignments resulted in non-conveyance to the Accident and Emergency department (A&E). On the other hand, studies describing the phenomenon from Specialist Ambulance Nurses’ (SAN) perspective [9–12] reveal patient safety issues and lack of clarity in the guidelines Furthermore, knowledge concerning the satisfaction patients and significant others when subjected to non-conveyance is relatively scare and mostly limited to qualitative studies [13–18]. The AS have been facing a growing demand due to changes in population demographics and an increasing number of calls and ambulance assignments [19]. Subsequently, over the years the AS have undergone changes from being a pure transport organisation, where standard procedures resulted in patients being conveyed to the A&E, to optimizing assessment and triage including referral to the A&E, primary care units or being discharged at the scene [11, 20]. To our knowledge, satisfaction with the care provided when patients are assessed as non-urgent and discharged at the scene is rarely described, indicating a possible knowledge gap and thus representing the rationale behind this study.

Due to national legislation, since 2005 ambulances in Sweden are staffed with a Registered Nurse (RN), who holds a three-year bachelor’s degree. The nurse is often teamed with another RN or an Emergency Medical Technician (EMT) [21]. In recent years Swedish ambulances are more commonly staffed with a SAN who holds a 1 year master degree in prehospital emergency care, thus a four-year university level education [22, 23]. RNs and SANs in the Swedish AS make medical decisions based on comprehensive guidelines and protocols [24]. In most Swedish counties, RNs and SANs can assess and refer patients to levels of care other than the A&E. The result of the assessment may e.g., be a non-conveyance decision at the scene or advice to contact a primary care unit [11, 24]. The levels of care in the Swedish health system are generally described as; self-care, primary care (including home visits by a local/regional GP) and the A&E at the hospital. Hereafter, the nurse in the ambulance will be referred to as a SAN, which is in line with the level of competence in the given context at the time of data inclusion.

Patient satisfaction is a multidimensional concept that is widely employed to evaluate e.g., waiting times, nursing skills [25, 26] and expectations of the provision of care in the A&E setting [27, 28]. Furthermore, patient satisfaction has been described as more than a lack of complaints about medical treatment [29], suggesting that care focused on medical or technical interventions does not lead to satisfied patients [30]. In general, research indicates that patients who are involved in their care and treated with dignity tend to be both more compliant and satisfied [31]. How patients experience care is a measure of its quality, which possibly has an impact on patient safety [32]. The source of dissatisfaction in the AS setting is often linked to poor communication, lack of a professional attitude, inadequate medical assessments, insufficient information and needs not being met by clinicians [13, 33, 34]. Patients express that they want to have their needs fulfilled, despite being aware that their condition is non-urgent and not life-threatening. In order to be reassured about symptoms such as pain, discomfort and anxiety, patients or their significant tend summon an ambulance [35, 36]. Consequently, the AS is often patients’ first contact with the health care system. Studies indicate that patients experience the care provided as positive [13, 37] and even expressed satisfaction [38, 39].

Many patients seeking care from the AS often present with conditions deemed non-urgent that could possibly be treated at a different level of care [35]. However, alerting the AS is often preceded on the part of the patient and the decision-making process leading to the actual call is not taken lightly [40]. Furthermore, study results have shown that patients have a need to be involved in their care and the decisions taken when being cared for and assessed by the SAN. Being taken seriously by the SAN and having their fear and anxiety alleviated have also been revealed as an important dimension that promotes autonomy and a sense of security [15, 17]. Significant others, defined as any person who is close and important to the patient [41], also play an important role as they provide both emotional and instrumental support to the patient [42]. In many cases a significant other is the one who phones for an ambulance [40], thus when a patient or a significant other calls for an ambulance they hope to gain control over their situation. Being a significant other often means responsibility and caring for the patient, which creates feelings of loneliness and vulnerability when illness occur [43]. In addition, significant others’ reasons for summoning an ambulance are powerlessness and their understanding the patient’s situation is urgent and that the affected person (i.e. the patient) might die. Therefore, significant others often feel relieved upon the SANs’ arrival [14, 16, 43]. Significant others have been found to experience their own form of suffering and are also in need of support. Taking care of and understanding the life world of significant others, i.e., their concerns, is a vital part of the SAN’s clinical operation [43]. There is limited knowledge of satisfaction among patients and significant others and whether AS staff have succeeded in optimizing assessments and decision-making in situations of non-conveyance to a healthcare facility. Measuring satisfaction with the care provided could possibly contribute to a better understanding of the expectations and demands of patients and significant others when patients are subjected to non-conveyance to the A&E and consequently discharged at the scene or referred to a primary care unit and/or general practitioner (GP).

## Aim

The aim of the study was to explore and compare satisfaction with the ambulance service among patients and significant others when the patient was discharged at the scene.

## Methods

### Design

The present study was designed as a cross-sectional, exploratory survey with a consecutive sample. The STROBE statement [44] was utilised to guide the preparation of this study and to ensure qualitative reporting based on the STROBE checklist for cross-sectional studies.

### Context, study setting and participants

A triage assessment tool called the Rapid Emergency Triage and Treatment System (RETTS) is commonly used nationwide in most AS and A&E organisations in Sweden. The RETTS triage system is intended as a prioritization tool to decide how quickly the patient needs to be assessed by a physician [45]. RETTS is based on the assessment of the patients’ major medical problem (flow charts entitled Emergency Symptoms and Signs, ESS) and considered to identify patients who are at risk of deteriorating at an early stage. The recording of vital parameters (VP) has been added to all RETTS flow charts, i.e. pulse rate, blood pressure, respiratory rate, oxygen saturation, body temperature and level of consciousness. The ESS codes include 58 different algorithms (2016 version) with the most common chief complaints. The combined assessed degree of urgency of both ESS and vital signs determines the priority level, which is indicated by the colours Red, Orange, Yellow, Green and Blue. The red level corresponds to a life-threatening situation, orange is potentially life-threatening, while yellow and green are non-urgent and blue represents a very limited medical risk. Thus, in many cases, the patients assessed as yellow, green or blue do not require conveyance to the A&E. An explanatory text that provides recommendations about what the assessing nurse should consider and what actions are recommended for each condition, such as taking an ECG for ongoing chest pain, is presented as a supplement to the flow chart. RETTS, combined with the clinical assessment, can be considered a prioritisation system [46, 47].

#### Setting

At the time of the study, some 250,000 inhabitants within the regional catchment area were served by the local AS, which operated 13 land-based ambulances depending on time of day and season. In 2018 the AS handled about 33,000 assignments (comprising all priority levels).

#### Participants

The participants were patients and significant others deemed non-urgent by the SAN upon arrival at the scene, thus discharged at the scene and not conveyed by ambulance to the A&E. The inclusion criteria were adult (18 years old or over), Swedish speaking and cognitively lucid. All participants who met the inclusion criteria were recruited consecutively within the catchment area by all SANs who handed out questionnaires to eligible participants. The participants were invited to take part in the study when the SANs were about to leave the patient’s home or accident scene, i.e., in conjunction with routine ambulance assignments.

### Data collection

In this exploratory study our intention was to amass 100 surveys for each group, i.e., patients and significant others respectively. All potential participants received an information letter that outlined the aim and details about the study, including the fact that the participants could withdraw at any time without stating reasons. A prepaid envelope addressed to the last author [A.R.] at Lund University, that could be dropped into any post box, was also included. Returning the completed survey was considered informed consent, which was stated in the information letter. As neither the questionnaire nor the envelope contained recognizable information, no reminder was sent. The data collection took place from June to September 2016 and was deemed complete when the target sample size was achieved.

#### Instrument

The survey included three parts; 1) Questions regarding demographics, 2) The SANs’ triage assessment (RETTS algorithm number and colour stated in the questionnaire before being handed out) and 3) The Consumer Emergency Care Satisfaction Scale (CECSS).

The CECSS is a questionnaire developed for measuring patient satisfaction in emergency care contexts [48, 49] and has been translated into Swedish and tested in A&E environments [50, 51] as well as in the AS [38, 39]. The instrument consists of 19 items with statements and a five-point Likert scale for measuring the response from 1 = completely disagree to 5 = completely agree. The CECSS measures satisfaction with nursing care including two dimensions: care (12 items) and discharge teaching (3 items) with the total score ranging from 15 to 75. Four negatively worded items are also included in the questionnaire to avoid/prevent response bias with the total score ranging from 4 to 20. A total score of 45 ≥ indicates satisfaction with care and < 45 indicates dissatisfaction, while in the negative items 12 ≤ indicates satisfaction and > 12 indicates dissatisfaction. The instrument has been evaluated for its psychometric properties in previous studies, with both the caring subscale and discharge teaching subscale demonstrating good reliability and internal consistency as well as adequate validity [49, 51]. Furthermore, two Swedish studies have investigated the provision of care by measuring patient satisfaction and the quality of the care received, where the CECSS was found to be feasible within the AS context [50, 51].

The CECSS has been further developed with design of a Swedish version that focuses on accompanying persons, i.e., significant others, the CECSS-A [52]. The CECSS-A consists of the same structure as the original version, i.e., 19 items and a five-point Likert scale. The modification of the original CECSS instrument involved slightly rephrasing four items to make them applicable to significant others. Another difference is that the CECSS-A has three subcategories; caring, teaching and in addition clinical competence [52]. Overall, the CECSS-A has shown satisfying psychometric properties, was deemed reliable and has proven feasible to use in the ED context to measure satisfaction among accompanying persons/significant others [52, 53].

### Statistical analysis

Demographic data of the survey participants, i.e., patients and significant others, were summarized by descriptive statistics. The Mann-Whitney U test was used for group comparison of the ordinal outcomes of individual items and Fisher’s exact test for group comparisons of the satisfaction score between patients and significant others. The sum scores scale was categorized into 15-44, 45-59 and 60-75, the negative items subscale was categorized into 4-8, 9-12 and 13-20. *P*-values < 0.05 were regarded as statistically significant. The statistical analyses were conducted using the Statistical Package for the Social Services (IBM SPSS Statistics for Windows, version 27.0.0).

## Results

A total of 507 questionnaires were handed out during the study period, of which 210 (41%) were returned; 110 from patients and 100 from significant others. Of these 48 were excluded due to incomplete responses. Hence, 162 complete questionnaires from 87 patients and 75 significant others were included and analysed. Of the included patients 49 were men and 37 were women, while the significant others comprised 43 men and 31 women. Their median age was 67 years and 62 years respectively. The characteristics of the patients and significant others are presented in Table 1.

**Table 1:** Characteristics of patients and significant others in the study (*n* = 162)

Variables of ambulance care	Patients (***n*** = 87)	Significant others (***n*** = 75)
**Gender (%)**		
Men	49 (56)	43 (58)
Women	37 (43)	31 (42)
**Age**		
Median (years) (Q1,Q3)	67 (47,79)	62 (48,72)
**Time of day** (%)		
08:00-17:00	48 (55)	37 (49)
17:00-24:00	22 (25)	25 (33)
24:00-08:00	17 (20)	11 (15)
**Assessed condition (%)**		
Medical	72 (44)	
Surgical	37 (23)	
Orthopaedic	31 (19)	
Other	5 (3)	
**Priority according to RETTS (%)**		
orange	9 (6)	
yellow	73 (45)	
green	61 (38)	

In total, 64% of the patients and significant others selected the most positive response alternatives on the CECSS. Similarly, on the four negatively worded items (i.e., reversed order), 77% selected the most positive alternatives. However, items concerning information/communication when discharged at the scene (i.e., not conveyed), such as “*The nurse gave me instructions about caring for myself/the patient at home”*,^*“*^*The nurse told me what problems to watch for”* and “*The nurse told me what to expect at home”* revealed that 19% respectively 17% of the patients and significant other were dissatisfied. One negatively worded item indicated that 18% were dissatisfaction, namely “*The nurse should have been more attentive than he or she was”.* Mean scores for the CECSS and the negatively worded items were 66.77 (SD 10.2) and 6.31 (SD 3.7) respectively. No significant differences were observed between patients and significant others on individual items. The distribution of responses on patient and significant others’ satisfaction for each item with the number and percentage for each response is presented in Table 2.

**Table 2:** Number and percentage distribution of CECSS item responses from patients (p) and significant others (so) (*n* = 162)

Item	p/so	N (%)				
		Total agreement				Total Disagreement
		5	4	3	2	1
1. The nurse performed her/his duties with skill	p	71 (81.6)	15 (17.2)	1 (1.1)	0 (0.0)	0 (0.0)
	so	60 (80.0)	12 (16.0)	1 (1.3)	2 (2.7)	0 (0.0)
2. The nurse seemed to know something about my/the patient’s illness or problem^a^	p	53 (60.9)	22 (25.3)	6 (6.9)	5 (5.7)	1 (1.1)
	so	54 (72.0)	13 (17.3)	6 (8.0)	1 (1.3)	1 (1.3)
3. The nurse knew what treatment I/the patient needed^a^	p	55 (63.2)	15 (17.2)	11 (12.6)	3 (3.4)	3 (3.4)
	so	46 (61.3)	15 (20.0)	10 (13.3)	1 (1.3)	3 (4.0)
4. The nurse gave me instructions about caring for myself/the patient at home^a^	p	53 (60.9)	16 (18.4)	4 (4.6)	5 (5.7)	9 (10.3)
	so	44 (58.7	11 (14.7)	7 (9.3)	6 (8.0)	7 (9.3)
5. The nurse should have been more attentive than he or she was	p	9 (10.3)	7 (8.0)	4 (4.6)	6 (6.9)	61 (70.1)
	so	11 (14.7	3 (4.0)	3 (4.0)	11 (14.7)	47 (62.7)
6. The nurse told me what problems to watch out for	p	53 (60.9)	12 (13.8)	8 (9.2)	7 (8.0)	7 (8.0)
	so	36 (48.0)	22 (29.3)	4 (5.3)	9 (12.0)	4 (5.3)
7. The nurse told me what to expect at home	p	42 (48.3)	14 (16.1)	12 (13.8)	6 (6.9)	13 (14.9)
	so	32 (42.7)	22 (29.3)	9 (12.0)	5 (6.7)	7 (9.3)
8. The nurse explained all procedures before they were done	p	63 (72.4)	12 (13.8)	4 (4.6)	6 (6.9)	2 (2.3)
	so	52 (69.3)	10 (13.3)	9 (12.0)	3 (4.0)	1 (1.3)
9. The nurse seemed too busy at the nurses’ station to talk to me	p	5 (5.7)	3 (3.4)	0 (0.0)	8 (9.2)	71 (81.6)
	so	6 (8.0)	2 (2.7)	2 (2.7)	10 (13.3)	55 (73.3)
10. The nurse explained things in terms I could understand	p	69 (79.3)	10 (11.5)	2 (2.3)	3 (3.4)	3 (3.4)
	so	59 (78.7)	9 (12.0)	3 (4.0)	2 (2.7)	2 (2.7)
11. The nurse was understanding when listening to my/the patient’s problem^a^	p	69 (79.3)	14 (16.1)	1 (1.1)	2 (2.3)	1 (1.1)
	so	63 (84.0)	8 (10.7)	3 (4.0)	1 (1.3)	0 (0.0)
12. The nurse seemed genuinely concerned about my pain, fear and anxiety	p	65 (74.7)	10 (11.5)	6 (6.9)	4 (4.6)	2 (2.3)
	so	56 (74.7)	13 (17.3)	2 (2.7)	2 (2.7)	2 (2.7)
13. The nurse was as gentle as he/she could be when performing painful procedures	p	64 (73.6)	9 (10.3)	5 (5.7)	4 (4.6)	5 (5.7)
	so	57 (76.0)	10 (13.3)	7 (9.3)	0 (0.0)	1 (1.3)
14. The nurse treated me as a number instead of as a person	p	8 (9.2)	5 (5.7)	4 (4.6)	7 (8.0)	63 (72.4)
	so	8 (10.7)	1 (1.3)	3 (4.0)	5 (6.7)	58 (77.3)
15. The nurse seemed to understand how I felt	p	60 (69.0)	18 (20.7)	5 (5.7)	2 (2.3)	2 (2.3)
	so	50 (66.7)	13 (17.3)	8 (10.7)	2 (2.7)	2 (2.7)
16. The nurse gave me a chance to ask questions	p	70 (80.5)	9 (10.3)	4 (4.6)	2 (2.3)	2 (2.3)
	so	61 (81.3)	7 (9.3)	6 (8.0)	0 (0.0)	1 (1.3)
17. The nurse was not very friendly	p	4 (4.6)	2 (2.3)	1 (1.1)	3 (3.4)	77 (88.5)
	sp	3 (4.0)	0 (0.0)	1 (1.3)	3 (4.0)	68 (90.7)
18. The nurse appeared to take time to meet my needs	p	68 (78.2)	14 (16.1)	2 (2.3)	0 (0.0)	3 (3.4)
	so	56 (74.7)	9 (12.0)	7 (9.3)	1 (1.3)	2 (2.7)
19. The nurse made sure that all my questions were answered	p	61 (70.1)	19 (21.8)	3 (3.4)	2 (2.3)	2 (2.3)
	so	54 (72.0	14 (18.7)	6 (8.0)	0 (0.0)	1 (1.3)

^a^Different phrasing of items to make them applicable to patients or significant others

There was no significant difference between the CECSS scores of patients and significant others, 94% in both groups indicated satisfaction (*p* = 0.87). Only five patients and four significant others reported dissatisfaction. Regarding the negatively worded items, 15% of the patients compared with 16% of the significant others indicated less satisfaction (*p* = 0.40) (Table 3).

**Table 3:** Comparison of CECSS scores and the CECSS negative items between patients and significant others

	Score	Patients (*n* = 87)	Significant others (*n* = 75)	*P* value
CECSS scores (15 items)^1^	75-60	71	59	0.87^3^
	59-45	11	12	
	44-15	5	4	
CECSS negative items (4 items)^2^	4-8	74	63	0.40^3^
	9-12	5	8	
	13-20	8	4	

^1^45 ≥ indicates satisfaction (i.e., patient scores of 3-5 on the 1-5 point Likert Scale)

^2^12 ≤ indicates satisfaction in the negative items

^3^Fisher’s exact test was used for comparison of the category scores

## Discussion

This study investigates and compares satisfaction with the AS among patients and significant others when the patient was discharged at the scene. The results show that patients and significant others were satisfied with the AS and the care provided by SANs when not conveyed to the A&E. No significant difference were observed between significant others and patients on the satisfaction scores.

Summoning an ambulance is not something that is taken lightly [40]. However, when the decision is made, patients expect a timely response in order to quickly alleviate anxiety due to the perceived illness [36]. This was also reflected in the present study as the participants indicated high scores on being *genuinely concerned about the patient’s or significant other’s pain, fear and anxiety*. Furthermore, it has been demonstrated that upon the arrival of the ambulance, the attenders values competence and skills of clinicians, who were perceived to be knowledgeable and thorough in their assessments [36], including obtaining a medical history as well as checking and measuring vital signs, e.g., blood pressure, pulse, oxygen saturation, respiratory rate, level of consciousness and body temperature [11]. If the patient is assessed as non-urgent and thereby not conveyed to the A&E, no major medical interventions or administration of medications are to be performed [12]. However, this study reveals that 17 – 19% of participants were dissatisfied regarding items on instructions about further care and what to expect when left at home. Providing information and explanations has been showed to be important in emergency service settings [16, 17, 27] and is associated with perceived satisfaction with the care provided [54]. This issue has room for improvement, i.e., indicating a need to emphasise satisfaction as positively associated with perceived interpersonal care [55], where staff members’ attentiveness to and gentleness in meeting patients’ needs, including provision of relevant information and instructions, should be highlighted [56]. In addition, in the case of non-conveyance, more interest should focused on what happens after patients or significant others are discharged at the scene, e.g. instructions regarding self-care advice [18]. As highlighted by SANs, the AS guidelines lack clarity and to a large extent do not include the patient perspective or assignments involving non-conveyance [9–12], which calls for further development.

The results from this study with high scores concerning listening and understanding the patients’ description of the perceived illness are consistent with previous studies on patients’ experiences of the AS and when subjected to non-conveyance [13]. Patients point out the basic human need to be taken seriously, which can be achieved by listening when they describe their illness experience, i.e., spending time with the patient [15]. This is in line with the results where both significant others and patients indicated that *The nurse appeared to take time to meet my needs* was important. A Swedish study reveals that ambulance assignments where patients are subjected to non-conveyance take 25 min on average [57]. Despite the relatively brief contact with the SAN, studies have shown that patients felt important and involved in their care [58], resulting in a sense of confirmation and confidence in their ability to handle the situation [15, 58].

Previous studies in the AS setting have found that before the arrival of the SANs significant others were feeling lonely and frightened, although forced to be present and care for the patient [43]. Therefore, significant others have a strong need to be de-burdened from the caring responsibility, which they assumed more or less involuntarily [14]. This was achieved temporarily when the SANs took over the interpretations of the symptoms, the responsibility and the decision-making from the significant other [14, 43]. This is in line with the present study, where allowing time to answer questions and explaining the situation in a clear manner was indicated as highly important.

Arguably, the issue of the patients and significant others’ trust in the SANs’ professionality could be considered a hugely influential factor [59], which probably affects satisfaction scores. As trust is dependent on multiple factors associated with person-centred care, such as patient participation, shared decision-making and the SANs’ credibility in the caring relationship, one can assume that perceptions of care and aligned decisions held by the patients as well as the significant others vary in line with their overall feeling of trust. As it can be assumed that the phenomenon of trust probably had a great impact on satisfaction scores in this study, measures to increase trust among all parties in the caring relationship should be considered central for achieving greater satisfaction with the provision of care [55, 60].

## Limitations

This study describes satisfaction among patients and significant others to patients in connection with the care of patients with non-urgent conditions and subjected to non-conveyance, which may complicate generalisation to all patient situations within the AS, including higher priority or potentially life-threatening conditions. A weakness of the study was the lack of non-Swedish speaking/reading participants, which potentially affected the study’s validity as the questionnaire used was only written in Swedish. Hence, the results could possibly be affected due to this lack of ethnic diversity. The result of a cross-sectional study should be interpreted with some caution as like most study designs, it has both advantages and disadvantages, e.g., it is not possible to draw conclusions about causality. The study was conducted within in a limited geographical area, which may also constitute a limitation. However, the chosen area contains a mix of rural and urban settings, as well as varying socioeconomic situations among the inhabitants. As the questionnaires were handed out by SANs in conjunction with ordinary ambulance assignments, selection bias cannot be ruled out, nor that the treatment provided was better than normal, resulting in higher satisfaction scores. However, the returned questionnaires contained varied responses, suggesting that they are representative. Finally, the response rate was reasonably high (41%), as a self-reported questionnaire typically demonstrates response rates of between 25 and 30% [61]. Here, one must consider whether those who responded were more satisfied with the care and more willing to complete the questionnaire, and if those who did not return the questionnaire were either dissatisfied and not willing to respond or satisfied but did not feel the need to respond. A number of returned questionnaires were excluded due to missing answers in the majority of the individual items, which we judged could be challenging to deal with in an appropriate statistical manner. Consequently, it cannot be completely ruled out that the result could be affected by this loss. Furthermore, as the sample is somewhat small, the generalisation of the findings may be limited.

## Conclusions

The results of this study reveal that overall, patients and significant others are satisfied with the care provided by the AS despite being discharged at the scene and thus not conveyed to the A&E. Satisfaction is an important outcome of nursing care that is associated with meeting the needs of patients and significant others. The participants in this study were especially satisfied with SANs’ interpersonal skills, e.g., making time and providing thorough information. However, guidelines for assignments involving non-conveyance, as well as information, instructions and what to expect when discharged at the scene need to be improved.

## Data Availability

The datasets generated and/or analysed during the current study are not publicly available since it was not acknowledged by the Regional Ethical Review Board at the time of study’s ethical vetting process. However, generated dataset and/or analysis are available from the corresponding author on reasonable request.

## Abbreviations

A&E: Accident and Emergency department
AS: Ambulance Service
CECSS: Consumer Emergency Care Satisfaction Scale
GP: General practitioner
VP: Vital Parameters
ESS: Emergency Symptoms and Signs
RN: Registered Nurse
EMT: Emergency Medical Technician
SAN: Specialist Ambulance Nurse
